# Long-Term Sequelae in Patients Treated for Recurrent CNS Relapses of Pediatric B-Cell Acute Lymphoblastic Leukemia: 20-Year Follow-Up, Neurological Consequences, and Survivorship Burden—A Case Report and Literature Review

**DOI:** 10.3390/pediatric18050121

**Published:** 2026-09-17

**Authors:** Maciej Niedźwiecki, Monika Lejman, Mieszko Czapliński, Janusz Springer, Anna Synakiewicz, Eliza Wasilewska

**Affiliations:** 1 Department of Paediatrics, Haematology and Oncology, Medical University of Gdańsk, Skłodowskiej-Curie 3a, 80-210 Gdansk, Poland; anna.synakiewicz@gumed.edu.pl; 2 Fahrenheit Union of Universities, Gdańsk Zwycięstwa Ave. 27, 80-219 Gdansk, Poland; 194058@uck.gda.pl (M.C.); janusz.springer@gumed.edu.pl (J.S.); eliza.wasilewska@gumed.edu.pl (E.W.); 3 Independent Laboratory of Genetic Diagnostics, Medical University of Lublin, A. Gębali 6, 20-093 Lublin, Poland; monika.lejman@umlub.pl; 4 Department of Genetic Diagnostics, Children’s University Hospital, A. Gębali 6, 20-093 Lublin, Poland; 5 University Clinical Centre, Medical University of Gdańsk, Dębinki 7, 80-952 Gdansk, Poland; 6 Division of Preventive Medicine & Education, Medical University of Gdansk, Skłodowskiej-Curie 3a, 80-210 Gdansk, Poland; 7 Department of Allergology, Medical University of Gdańsk, Skłodowskiej-Curie 3a, 80-210 Gdansk, Poland

**Keywords:** acute lymphoblastic leukemia, relapse, children, cytarabine, pediatric acute lymphoblastic leukemia, central nervous system relapse, liposomal cytarabine, intrathecal chemotherapy, allogeneic hematopoietic stem cell transplantation, neurotoxicity, survivorship

## Abstract

Central nervous system (CNS) relapse remains a major cause of treatment failure in pediatric acute lymphoblastic leukemia (ALL). Repeated isolated CNS relapse is particularly rare and associated with poor prognosis, while optimal therapeutic strategies remain insufficiently defined. Intensified CNS-directed therapy may improve disease control but is also associated with substantial long-term neurotoxicity and survivorship burden. We present the case of a boy with favorable-risk B-cell ALL who developed two isolated CNS relapses despite a good initial response to frontline therapy and absence of classical CNS relapse risk factors. The second relapse was associated with extensive meningeal involvement and optic nerve infiltration. The patient underwent intensive multimodal CNS-directed therapy, including repeated intrathecal chemotherapy, liposomal cytarabine administered according to the IntReALL 2010 protocol, cranial irradiation, and allogeneic hematopoietic stem cell transplantation (alloHSCT) from a matched sibling donor. Durable long-term remission was achieved despite the extremely unfavorable prognosis that is associated with a second isolated CNS relapse. A twenty-year follow-up extending into early adulthood revealed substantial late complications, including epilepsy, transient ischemic attack, optic nerve injury, endocrinopathies, obesity, secondary thyroid malignancy, neurocognitive difficulties, and depression requiring long-term psychiatric and psychological support. The present case illustrates the complex balance between effective CNS disease control and cumulative treatment-related neurotoxicity in pediatric ALL survivors. It also highlights the cumulative CNS injury and long-term survivorship burden that is associated with repeated CNS relapse and multimodal CNS-directed therapy.

## 1. Introduction

Relapse of acute lymphoblastic leukemia (ALL) remains a major clinical challenge in children who are treated with contemporary therapeutic protocols. Despite substantial improvement in survival rates over recent decades, recurrent disease continues to represent the leading cause of treatment failure in pediatric ALL [1,2]. The prognosis after relapse depends mainly on the timing and location of recurrence. Isolated late extramedullary relapses are generally associated with more favorable outcomes than early bone marrow relapses [2,3,4].

In Poland, approximately 200–250 children are diagnosed with ALL annually, and nearly 90% of patients achieve long-term remission without relapse [1]. Consequently, a first relapse of ALL is currently diagnosed in only approximately 20–30 pediatric patients per year nationwide. Repeated isolated CNS relapses therefore represent exceptionally rare and clinically challenging events in pediatric hemato-oncology practice.

The CNS is considered to be a sanctuary site in ALL because penetration of systemic chemotherapy through the blood–brain and blood–CSF barriers remains limited. As a result, residual leukemic cells may survive within the CNS despite apparently effective systemic treatment [3]. Particularly challenging are patients who develop repeated CNS relapse despite initially favorable-risk disease characteristics and good early response to frontline therapy.

The prognosis after a second isolated CNS relapse is particularly unfavorable, and the therapeutic strategies in this setting remain insufficiently defined [4,5]. In such patients, treatment decisions often require balancing effective CNS disease control against the risk of long-term neurotoxicity. Achieving durable remission frequently requires highly intensive multimodal therapy, which may itself become a major source of long-term morbidity.

Treatment intensification in patients with recurrent CNS disease commonly involves repeated intrathecal chemotherapy, CNS irradiation, and hematopoietic stem cell transplantation (HSCT). However, these approaches are associated with substantial acute and delayed toxicity, particularly affecting the developing central nervous system [2,6,7,8]. Combined exposure to repeated CNS-directed therapies may lead to long-term neurological, vascular, endocrine, neurocognitive, visual, and psychosocial complications.

One proposed approach to improve CNS disease control is the use of liposomal formulations of cytotoxic agents. Liposomal cytarabine was developed to prolong drug half-life within the cerebrospinal fluid (CSF), enhance CNS drug distribution, and maintain sustained cytotoxic activity in the meningeal compartment [9,10]. Compared with conventional cytarabine formulations, liposomal cytarabine enables prolonged therapeutic CSF concentrations and improved drug distribution within the CNS compartment [9,10]. In selected high-risk patients with recurrent CNS disease, this strategy may facilitate temporary CNS disease control and potentially enable subsequent consolidation with allogeneic hematopoietic stem cell transplantation (alloHSCT).

When the patient described herein was treated, the role of alloHSCT in children with isolated CNS relapse remained controversial, and standardized therapeutic approaches were not clearly established [5,11]. While some treatment groups supported intensive chemotherapy alone in selected patients with local relapse, others considered consolidation with alloHSCT to be justified in particularly high-risk clinical scenarios. This therapeutic uncertainty further emphasizes the clinical relevance of the present case and its exceptionally long-term follow-up. Nevertheless, prolonged CNS exposure to intrathecal chemotherapy may also increase the risk of neurotoxicity, including seizures, encephalopathy, arachnoiditis, and delayed neurological injury [7,8]. Long-term data describing neurological and psychosocial outcomes in survivors who were treated with repeated CNS-directed therapy remain limited, particularly in pediatric patients.

The aim of this report was to present a rare case of a second isolated CNS relapse in pediatric B-cell acute lymphoblastic leukemia that was successfully treated with intrathecal liposomal cytarabine and alloHSCT from a matched sibling donor, as well as to discuss the long-term neurological sequelae that are associated with intensive multimodal CNS-directed therapy.

## 2. Case Report

### 2.1. Primary Disease

The initial diagnosis was made in a 6-year-old boy in the Department of Paediatrics, Haematology, Oncology and Endocrinology of the Medical University of Gdańsk. The laboratory tests showed significant anemia (Hb = 10.21 g%), leucopoenia (WBC = 2.62/mm^3^), and thrombocytopenia (PLT = 104,000/mm^3^). The boy complained of generalized weakness and pain. Physical examination revealed skin pallor, generalized lymphadenopathy, and hepatosplenomegaly. Based on the cytomorphological, immunophenotypic, cytogenetic, and cytochemical examination of the collected bone marrow, acute lymphoblastic leukemia of the common type with favorable prognostic factors was diagnosed. Clinical imaging revealed no extramedullary lesions. The boy was treated in accordance with the ALL-IC 2002 [12] program intended for a standard risk group. No severe toxicities during treatment that would imply a delay of subsequent chemotherapy cycles were reported.

### 2.2. First ALL Relapse Within the CNS

The first ALL relapse within the CNS was diagnosed 37 months after the initial diagnosis. The boy complained of headaches, dizziness, and double vision. An MRI of the head and a neurological examination showed no deviations from the normal condition. Only the lumbar puncture revealed 1370 lymphoblasts/μL of cerebrospinal fluid. The boy was diagnosed with late isolated cerebral relapse. He was qualified into the S1 risk group according to the Berlin–Frankfurt–Münster group stratification [13]. ALL relapse therapy was introduced in the same month. CNS remission, i.e., the elimination of cerebrospinal fluid (CSF) blasts, was achieved in the R2 course. This treatment stage induced numerous infectious complications, including invasive pulmonary mycosis, requiring a long withdrawal from therapy. After the 5th chemotherapy course, the boy experienced a seizure of unknown etiology. Neither ophthalmic and neurological examinations nor clinical imaging (head CT) revealed any perceptible deviations from the normal condition. Finally, the seizure was explained by fluid and electrolyte metabolism disorders resulting from chemotherapy. Intensive chemotherapy was finished 6.5 months later. Six months after the relapse diagnosis the boy started craniospinal irradiation therapy at a dose of 18 Gy, which was completed as planned. Maintenance therapy ended 4.5 years after the initial diagnosis according to protocol.

### 2.3. Second ALL Relapse Within the CNS

The boy began to experience severe headaches 6 months after the end of the first relapse therapy. The initial fundus examination and EEG showed no alarming changes. Additional tests performed 14 days later revealed fundus stasis and an MRI showed thickening of the cerebrospinal meninges. Blastic pleocytosis was found in the CSF, confirming the suspicion of another CNS relapse. After consultation with the national coordinator for ALL treatment in children, we started chemotherapy according to the IntReALL 2010 [14] protocol with intrathecal liposomal cytarabine. The boy also qualified for bone marrow transplantation from a family donor (brother). This decision was made based on the particularly poor prognosis that is associated with a second isolated local relapse. We maintained intrathecal liposomal cytarabine administration, but, due to exacerbating neurological symptoms appearing to result from treatment, we withdrew from further intrathecal chemotherapy. The child experienced periodic paroxysmal headaches of unknown etiology, accompanied by vegetative symptoms in the form of anxiety, tachycardia, severe sweating, and malaise. Complications of intravenous chemotherapy also involved steroid-induced Cushing’s syndrome with diabetes and hypertension, subclinical hypothyroidism, hypercortisolism, and hyperinsulinism.

During implementation of the IntReALL 2010 protocol, the patient received 6 doses of liposomal cytarabine, 35 mg each (administered intrathecally) with steroid cover (intrathecal prednisone, 10 mg). After the 6th dose, the boy experienced alarming symptoms: increasing headaches and severe generalized seizures. Clinical imaging and ophthalmologic and neurologic consultations were performed to exclude another relapse of the primary disease. No leukemic cells were found in the CSF. The ophthalmic exam revealed persistent fundus stasis in both eyes, while the head EEG, MRI, and CT showed no features of relapse. Considering the above, the cytarabine therapy was abandoned; the reported symptoms seemed to be related to complications of intrathecal chemotherapy and radiation therapy carried out after chemical treatment of the first cerebral relapse.

### 2.4. Subsequent Course

Six months after receiving allo-BMT from his brother the boy was followed by the Department of Paediatrics, Haematology, Oncology and Endocrinology of the Medical University of Gdańsk until he reached adulthood. During the observation period he still experienced periodic headaches and dizziness, but weakness and malaise were much rarer. After consulting a pediatric neurologist and increasing his oxcarbazepine dose to 2×900 mg, with the inclusion of i.v. clonazepam (2×0.5 mg), which was gradually discontinued, the generalized seizures ceased for a while. His neurological history is still ongoing. He experiences episodes of generalized epileptic seizures (last hospitalization due to that cause took place this year, 20 years after the initial diagnosis). The patient also has a parasagittal meningioma (left frontal region) and a vascular malformation in the left occipital lobe. Three years ago, he had an episode of transient ischemic anemia too. His right optic nerve is permanently damaged with partial loss of vision.

In addition the patient was diagnosed with another neoplasia, papillary thyroid cancer, which was treated by thyroidectomy 11 years ago, followed by adjuvant radioiodine therapy. Ten years ago he underwent closure of the Botallo duct. He remains under the care of outpatient departments in hematology and endocrinology. The chemotherapy, radiotherapy and complications after allo-BMT caused further side effects: hypergonadotropic hypogonadism and partial insufficiency of anterior hypophysis. He also suffers from obesity, diabetes mellitus, mixed dyslipidemia, hypertriglyceridemia (in the course of which he had two episodes of oedematous acute pancreatitis, 6 years ago and 1 year ago). Currently the patient is 25 years old. Despite the challenges and a long list of medical conditions he graduated from high school and is currently enrolled at university. Due to his health issues, he struggles with depression.

**Figure 1:**
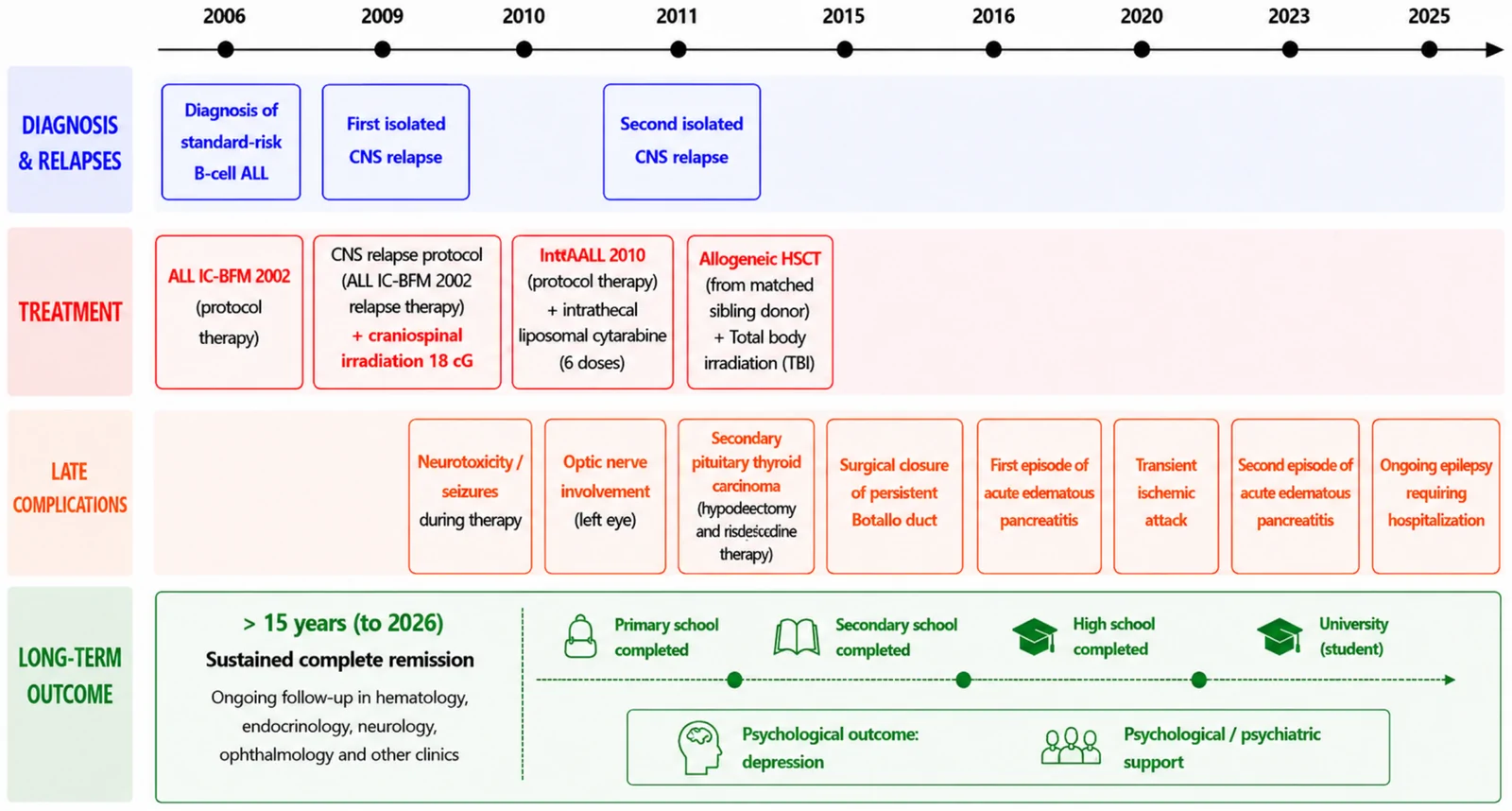
Timeline of disease course, CNS-directed therapy, late complications, and long-term survivorship following two isolated CNS relapses of B-cell acute lymphoblastic leukemia.

The detailed timeline of the disease course, therapy, and complications is shown in Figure 1.

## 3. Discussion

The present case illustrates several uncommon and clinically important aspects of pediatric B-cell acute lymphoblastic leukemia. The patient developed two isolated CNS relapses despite initially favorable-risk disease characteristics and a good response to frontline therapy. Repeated CNS recurrence occurred in the absence of classical high-risk features that are typically associated with CNS relapse. Despite the extremely unfavorable prognosis after a second isolated CNS relapse, durable long-term remission was ultimately achieved. At the same time, successful CNS disease control was accompanied by substantial long-term neurological, vascular, endocrine, visual, metabolic, and psychosocial sequelae related to cumulative CNS-directed therapy. The case therefore reflects the difficult balance between CNS sanctuary-site biology, aggressive salvage therapy, and long-term survivorship burden in pediatric ALL.

### 3.1. Risk Factors and Epidemiology of Cerebral ALL Relapses

Despite major advances in contemporary therapeutic protocols, relapse remains the leading cause of treatment failure in childhood acute lymphoblastic leukemia (ALL) [3,5]. CNS relapse occurs in approximately 3–10% of pediatric ALL patients and continues to represent a clinically challenging form of recurrence [3]. Prognosis is generally more favorable in children with late isolated CNS relapse occurring more than 18 months after initial diagnosis and treatment completion [2,3]. Nevertheless, repeated isolated CNS relapse remains associated with poor long-term outcomes [4].

The biological mechanisms underlying CNS relapse in pediatric ALL are still not fully understood. One of the most important recognized risk factors is traumatic lumbar puncture at diagnosis, especially in patients with high peripheral blast counts [3,15,16,17]. CNS-2 status and insufficient CNS-directed therapy have also been associated with increased risk of CNS recurrence in some studies, although the prognostic significance of CNS-2 remains controversial [18].

The established risk factors for CNS relapse include T-cell immunophenotype, hyperleukocytosis, unfavorable cytogenetic abnormalities such as t(9;22) and t(4;11), and initial CNS involvement [3,4]. Additional reported risk factors include male sex, hepatomegaly, CNS-2 status, and age below 2 years or above 6 years [15,16,19]. However, none of these classical risk factors were clearly present in the described patient.

The occurrence of repeated CNS relapse despite the absence of overt CNS involvement at initial diagnosis may support the concept of occult leukemic persistence within CNS sanctuary sites. Limited sensitivity of standard diagnostic methods may allow subclinical leukemic infiltration to remain undetected during frontline therapy. In our patient, optic nerve involvement and meningeal disease may additionally suggest persistence of leukemic cells within anatomically protected CNS compartments characterized by reduced penetration of systemic chemotherapy.

Recent studies have also suggested that individual pharmacogenetic variability may influence CNS relapse risk. Polymorphisms affecting vitamin D receptor pathways, cytochrome P450 activity, P-glycoprotein regulation, or methotrexate metabolism may potentially contribute to reduced efficacy of CNS prophylaxis in selected patients [20]. Although such mechanisms remain speculative in the present case, they may partially explain the occurrence of repeated CNS relapse despite otherwise favorable clinical characteristics.

Severe infectious and toxic complications occurring during treatment of the first CNS relapse resulted in substantial prolongation and modification of therapy. This may also have contributed to insufficient eradication of residual leukemic cells within the CNS compartment.

Potential mechanisms that may have contributed to repeated isolated CNS relapse in the present patient are summarized in Table 1.

### 3.2. Challenges in Treatment of Second Isolated CNS Relapse

A second isolated CNS relapse in pediatric ALL represents a particularly difficult therapeutic scenario that is associated with a poor prognosis and limited evidence regarding optimal salvage strategies [4,5,21]. Prognosis after a second CNS relapse is closely related to the time from primary diagnosis to relapse, as well as relapse location. Patients with late isolated CNS relapse generally achieve better outcomes than patients with early bone marrow recurrence; however, repeated isolated CNS relapse still carries extremely unfavorable prognosis [2,4,21].

The present case was particularly challenging because another CNS relapse occurred only 6 months after completion of maintenance therapy despite rapid achievement of remission during treatment of the first CNS relapse. Extensive meningeal involvement and optic nerve infiltration may have represented important adverse prognostic factors contributing to repeated CNS recurrence. Clinical manifestations such as double vision and imaging abnormalities involving the optic nerve suggested leukemic infiltration within anatomically protected CNS compartments characterized by limited penetration of systemic chemotherapy. Similar limitations may also apply to therapeutic irradiation, which may not uniformly eradicate residual leukemic cells in selected sanctuary regions [22].

One strategy aimed at overcoming limited CNS drug penetration involves intensification of intrathecal therapy and the use of modified cytotoxic formulations with improved pharmacokinetic properties [23]. Liposomal cytarabine was developed to prolong drug half-life within the cerebrospinal fluid and maintain sustained cytotoxic concentrations within the meningeal compartment [9,10]. Compared with conventional cytarabine, liposomal formulations may provide more effective drug distribution within the CNS and prolonged exposure of leukemic cells to cytotoxic therapy [9,10]. Liposomal cytarabine can even be detected in cerebrospinal fluid by flow cytometry long after administration [24].

Several pediatric studies demonstrated promising efficacy of intrathecal liposomal cytarabine in leukemic CNS relapse, although treatment-related neurotoxicity remains an important limitation [21,25,26,27,28]. In our patient, intrathecal liposomal cytarabine was introduced during treatment according to the IntReALL 2010 protocol in an attempt to improve CNS disease control before alloHSCT. In retrospect, this strategy may have contributed to temporary stabilization of CNS disease before transplantation. However, at the time when the patient was treated, the balance between potential therapeutic benefit and treatment-related neurotoxicity remained highly uncertain.

An important clinical dilemma involved the decision to perform alloHSCT after a second isolated CNS relapse. The therapeutic approaches differed substantially between the treatment groups, and standardized recommendations were lacking [5,11]. Some groups supported intensive chemotherapy alone in selected patients with local relapse, whereas others considered transplantation to be justified in patients with particularly high-risk disease characteristics. In our patient, repeated CNS relapse, optic nerve involvement, meningeal infiltration, and progressively shorter relapse-free intervals strongly supported treatment intensification with alloHSCT from a matched sibling donor.

A second therapeutic strategy described in the literature involves intraventricular chemotherapy administered through ventricular access devices [3,29]. Nevertheless, all intensified CNS-directed treatment modalities are associated with substantial toxicity risk. The present case illustrates the difficult balance between aggressive salvage therapy required to achieve durable remission and the risk of cumulative long-term CNS injury. At that stage of treatment, concerns regarding late complications seemed secondary to the extremely poor prognosis that is associated with a second isolated CNS relapse.

The present case also reflects the evolving concept of CNS-directed salvage therapy in pediatric ALL. While intensified multimodal treatment strategies may contribute to durable long-term remission even in extremely high-risk clinical scenarios, they may simultaneously increase the risk of delayed neurological, endocrine, vascular, and psychosocial sequelae observed in long-term survivors.

### 3.3. Neurotoxicity and Long-Term Complications

An important challenge that is associated with intensified CNS-directed therapy in pediatric ALL involves the risk of acute and delayed neurotoxicity. In patients with repeated CNS relapse, cumulative exposure to intrathecal chemotherapy, cranial irradiation, total body irradiation (TBI), and alloHSCT may result in substantial long-term neurological and systemic morbidity; leukemia itself is also a strong risk factor as it frequently involves the central nervous system causing severe effects on its functioning.

In the present case, interpretation of individual treatment-related complications remains particularly difficult because the patient was exposed to multiple sequential CNS-directed interventions over several years. Intensive intrathecal chemotherapy, cranial irradiation administered during treatment of the first CNS relapse, and TBI conditioning before alloHSCT likely contributed synergistically to the observed late complications.

Cranial irradiation is considered to be one of the major risk factors for delayed neurological injury in survivors of childhood ALL. Radiation-induced vascular damage, white matter injury, neurocognitive dysfunction, endocrinopathies, and secondary malignancies have all been widely described in the literature [7,8]. Similarly, repeated intrathecal chemotherapy may contribute to chemical arachnoiditis, seizures, leukoencephalopathy, and delayed neurotoxicity, particularly in heavily pre-treated patients [7,8].

The spectrum of late complications observed in our patient included epilepsy, transient ischemic attack, optic nerve injury, neurocognitive difficulties, endocrinopathies, obesity, secondary thyroid malignancy, and depression requiring long-term psychiatric and psychological support. These complications likely did not result from a single therapeutic modality but rather cumulative CNS injury associated with repeated leukemic CNS involvement combined with sequential multimodal CNS-directed therapy.

The present case therefore illustrates the complex balance between achieving durable CNS disease control and limiting long-term survivorship burden. Although intensified salvage therapy contributed to long-term remission, it was also associated with substantial neurological, vascular, endocrine, visual, metabolic, and psychosocial sequelae persisting into early adulthood.

**Figure 2:**
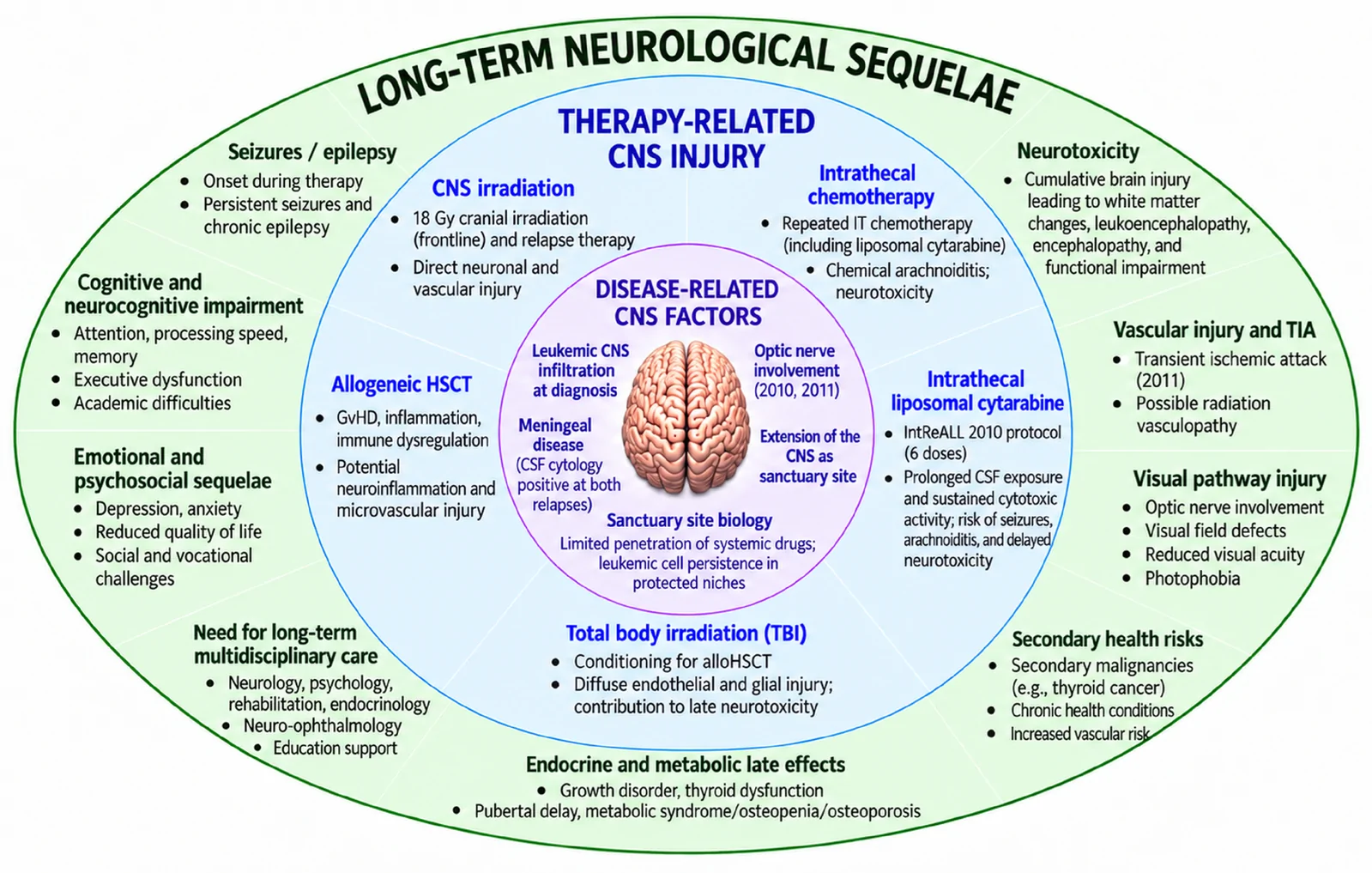
Conceptual model of cumulative CNS injury associated with repeated isolated CNS relapse and multimodal CNS-directed therapy in pediatric B-cell acute lymphoblastic leukemia.

This cumulative injury model, its relation with the therapy mode, and the potential effects on long-term toxicities are summarized conceptually in Figure 2.

### 3.4. Genetic and Pharmacogenetic Determinants of Relapse and Treatment-Related Toxicity

Beyond the clinical and anatomical factors discussed above, host and tumor genetics may help to explain why this patient, classified as favorable-risk and lacking classical CNS risk features, experienced repeated CNS recurrence together with an unusually severe burden of treatment-related complications. Contemporary genomic studies indicate that relapse risk in childhood ALL is strongly subtype-dependent and a substantial proportion of relapses arise in patients who are initially classified as standard- or favorable-risk. In a large Children’s Oncology Group analysis, specific genomic subtypes and cooperating lesions, such as PAX5-altered ALL and IKZF1plus, were independently associated with relapse despite otherwise favorable presenting features [30]. Although the leukemic genome of the present patient was not characterized at the molecular resolution that is available today, this observation supports the possibility that an adverse genomic lesion was present beneath a clinically favorable phenotype and contributed to repeated recurrence.

The propensity for isolated CNS relapse, in particular, may reflect intrinsic biological properties of the leukemic clone rather than conventional risk factors alone. Preclinical and translational data indicate that CNS infiltration in B-cell precursor ALL is driven by molecular programs governing leukemic entry into, and survival within, the leptomeningeal and perivascular niche, including adhesion and cytokine-receptor signaling, such as IL7R, and the capacity of dormant leukemic cells to persist in a sanctuary microenvironment with limited drug penetration [31]. These mechanisms are consistent with the concept of occult CNS persistence proposed earlier and could account for repeated meningeal and optic nerve involvement in a patient without overt CNS disease at initial diagnosis. Constitutional genetic background may further modulate susceptibility; for instance, rare deleterious variants in DNA-repair genes such as NBN have recently been associated with predisposition to childhood B-ALL [32], illustrating how the germline can shape both leukemogenesis and the response of normal tissues to genotoxic therapy.

Inherited pharmacogenetic variation offers a plausible mechanistic link between the patient’s genotype and the severity of CNS-directed treatment toxicity. Inter-individual differences in folate metabolism and drug-transporter function, mediated by polymorphisms in genes including MTHFR, SLCO1B1, ABCB1 and ABCC2, modulate systemic and central nervous system exposure to methotrexate and other cytotoxic agents and have been associated both with reduced efficacy of CNS-directed prophylaxis and treatment-related neurotoxicity [33]. Variants that delay drug clearance or alter cerebrospinal fluid pharmacokinetics may simultaneously diminish leukemic eradication within the CNS and increase the risk of seizures, encephalopathy and arachnoiditis of the kind observed in this patient. Although the pharmacogenetic profile of the present patient is unknown, such mechanisms may partly explain the coexistence of insufficient CNS disease control and a pronounced neurotoxic response to repeated intrathecal and systemic therapy.

Finally, genetic susceptibility is increasingly recognized as a determinant of late effects and subsequent neoplasms, which is particularly relevant given this patient’s development of papillary thyroid carcinoma and a parasagittal meningioma after cranial irradiation, total body irradiation and radioiodine therapy. Large survivorship cohorts have demonstrated that germline cancer-predisposing variants, pathogenic variants in DNA-repair genes and polygenic risk interact with radiotherapy and chemotherapy exposures to substantially increase the risk of subsequent neoplasms; notably, elevated genetic risk has been estimated to account for a major fraction of radiation-associated thyroid cancers and contribute to meningioma risk among childhood cancer survivors [34]. This convergence of cumulative treatment exposure and constitutional susceptibility provides a coherent framework for the multiplicity of late complications observed here.

Taken together, these considerations suggest that, in retrospect, integrated germline and somatic genomic profiling combined with pharmacogenetic-guided dosing might have refined both relapse-risk stratification and toxicity prevention in this patient. Although they remain hypothesis-generating in the present historical case, in which such analyses were not available, they underline the potential value of incorporating germline and tumor genetic testing, together with pharmacogenetic screening, into the management and long-term surveillance of children with high-risk or repeatedly relapsing ALL.

### 3.5. Literature Review of Published Pediatric Cases

To place our patient in a broader clinical context, we reviewed published pediatric reports describing the use of intrathecal liposomal cytarabine in CNS relapse of acute lymphoblastic leukemia. The PubMed database was searched using the following query:

(((leukaemia) AND (intrathecal)) AND (liposomal)) AND (cytarabine)

The search identified 39 publications. After exclusion of duplicate records, non-English articles, adult studies, animal models, and reports that were not directly related to pediatric CNS relapse treatment, six pediatric publications were selected for qualitative comparison (Table 2).

Most published reports described the use of liposomal cytarabine in patients with recurrent or refractory CNS leukemia treated with intensified CNS-directed therapy [21,25,26,27,28]. The analyzed studies differed substantially with regard to patient characteristics, extent of CNS involvement, treatment protocols, concomitant therapies, and reported outcomes. Nevertheless, several authors reported good local CNS disease control associated with prolonged cerebrospinal fluid exposure achieved with liposomal formulations [9,10,24,25,26,27,28].

At the same time, neurotoxicity remained an important limitation of treatment. The reported adverse events included headache, arachnoiditis, seizures, encephalopathy, and delayed neurological complications, particularly in heavily pre-treated patients receiving combined CNS-directed modalities [7,8,25,26,27,28]. It is of vital importance to note that the toxicity and side effects are multifactorial. It is unclear and unjustified to attribute all these effects solely to cytarabine. Also, in the aforementioned literature, no clear data on the significance of the role of cytarabine in developing these effects was given.

Most available reports focused mainly on short-term treatment response and acute toxicity. Long-term follow-up data describing neurological, endocrine, vascular, neurocognitive, and psychosocial sequelae remain scarce.

Compared with previously published pediatric cases, our patient represents unusually long-term follow-up after a second isolated CNS relapse treated with intrathecal liposomal cytarabine and alloHSCT. The case additionally illustrates the cumulative neurological and psychosocial burden that is associated with repeated CNS relapse and multimodal CNS-directed therapy.

The case shows several uncommon and clinically important aspects of pediatric B-cell acute lymphoblastic leukemia. The patient developed two isolated CNS relapses despite initially favorable-risk disease characteristics and a good response to frontline therapy. Repeated CNS recurrence occurred in the absence of classical high-risk features that are typically associated with CNS relapse. Durable long-term remission was achieved despite an extremely unfavorable prognosis after a second isolated CNS relapse. Finally, successful CNS disease control was accompanied by substantial long-term neurological, vascular, endocrine, visual, metabolic, and psychosocial sequelae resulting from cumulative CNS-directed therapy.

The case therefore highlights the complex interplay between CNS sanctuary-site biology, multimodal salvage therapy, and long-term survivorship burden in pediatric ALL.

## 4. Conclusions and Clinical Implications

The present case illustrates the rare occurrence of repeated isolated CNS relapse in a patient with initially favorable-risk pediatric B-cell acute lymphoblastic leukemia and a good response to frontline therapy. Despite the extremely unfavorable prognosis that is associated with a second isolated CNS relapse, durable long-term remission was achieved using intensive multimodal CNS-directed therapy, including intrathecal liposomal cytarabine and alloHSCT from a matched sibling donor.

From the current perspective, intrathecal liposomal cytarabine may have contributed to temporary CNS disease control before transplantation, although the exact impact of individual treatment modalities cannot be clearly separated in such heavily pre-treated patients.

At the same time, the case highlights the substantial long-term survivorship burden that is associated with repeated CNS relapses and cumulative CNS-directed therapy. The observed neurological, endocrine, vascular, visual, cognitive, metabolic, and psychosocial sequelae likely resulted from the combined effects of repeated leukemic CNS involvement, intrathecal chemotherapy, CNS irradiation, total body irradiation, and alloHSCT. The problem of long-term toxicities of chemotherapeutic treatments is by no means limited to our case; increased efficiency of chemotherapy and longer survival of patients who are treated with modern chemotherapeutics inevitably result in increased risk of long-term toxicities. The current attempts at precise estimation of these causes resulted in the introduction of new parameters that are used to analyze them. Severe toxicity-free survival (STFS) was introduced [35]. This parameter analyzes not only the survival and cancer-free periods but also whether serious treatment side effects occurred in the patient. There is a list of 21 severe side effects (e.g., blindness) that are taken into account [35]. Especially in a case like ours, this indicator would be of significant relevance.

Our observations emphasize the need for prolonged multidisciplinary follow-up in survivors who are treated with intensified CNS-directed salvage therapy and underline the importance of balancing effective CNS disease control with the risk of cumulative long-term neurotoxicity.

## Figures and Tables

**Table 1:** Potential mechanisms contributing to repeated isolated CNS relapse in the present case.

Potential Mechanism	Possible Relevance in the Present Case
Sanctuary-site biology	Possible persistence of leukemic cells within CNS compartments
Optic nerve involvement	Limited penetration of systemic chemotherapy
Meningeal infiltration	Diffuse CNS disease distribution
Treatment delays	Prolongation of therapy after infectious complications
Limited sensitivity of initial diagnostics	Potential occult CNS disease

**Table 2:** Summary of the literature.

	Group Characteristics			Therapy Characteristics		Effects	Side Effects
	N	Median Age (y)	Age Range (y)	Dose	N of Cycles		
[10]	9	7	–	–	6	–	Headache, vomiting, backache
[21]	18	10	4–19	25 → 35 → 50 mg	11	CR 4; PR 3; SD 2; PD 5	Arachnoiditis, headache
[25]	30 (21 male, 9 female)	9.4	0.8–18	Age-dependent dosing from 20 to 50 mg per cycle	Median 4 doses (2–9)	CNS CR 25	PRES (1); strabismus and clonus in the lower right limb (1); partial seizures due to hemorrhagic stroke in aplastic phase (1); aphasia, ataxia, left hyposthenia, and chorea (1)
[26]	4	–	0.3–17	25 mg (2), 35 mg (2)	2–4	CR 4	Pseudotumor cerebri (1), irritability (1)
[27]	6 (5 male, 1 female)	11	2.5–16	25 → 35 → 50 mg	Median 6 (3–7)	CR 7	No side effects; one case of mild headache, but all patients died (3 cases DOD, 2 after HSCT, and 1 from septic shock)
[28]	3 (2 male, 1 female)	–	5–18	20 → 35 → 50 mg	Median 3 (1–9)	CR 3	Drowsiness, agitation, disorientation, hallucinations (grade III), mild headache, and seizures

## Data Availability

Basic data is provided within the manuscript. Other data is available on request. In order to obtain this data, contact the corresponding author.

## Abbreviations

The following abbreviations are used in this manuscript.

ALL	Acute Lymphoblastic Leukemia
CNS	Central Nervous System
BMT	Bone Marrow Transplantation
WBC	White Bloodcell Count
HSCT	Hematopoietic Stem Cell Transplant
PLT	Platelet Count
IntReALL 2010	International Study for Treatment of Standard Risk Childhood Relapsed ALL 2010—A Randomized Phase III Study Conducted by the Resistant Disease Committee of the International BFM Study Group
ALL-IC 2002	A Randomized Trial of the I-BFM-SG for the Management of Childhood non-B Acute Lymphoblastic Leukemia (ALL IC-BFM 2002)
EEG	Electroencephalography
MRI	Magnetic Resonance Imaging
CT	Computer Tomography
CR	Complete Remission
PR	Partial Remission
SD	Stable Disease
PD	Progression of Disease
PRES	Posterior Reversible Encephalopathy Syndrome
